# Cortical response to transient and long-term visual field loss

**DOI:** 10.1093/cercor/bhaf237

**Published:** 2025-09-09

**Authors:** Marco Ninghetto, Georgios A Keliris, Kamil Szulborski, Tomasz Gałecki, Bartosz Kossowski, Daan Panneman, Frans P M Cremers, Monika Ołdak, Jacek P Szaflik, Kalina Burnat

**Affiliations:** Nencki Institute of Experimental Biology, PAS, 3 Pasteur Street, 02-093 Warsaw, Poland; Department of Neurology, Brigham and Women’s Hospital Harvard Medical School Boston, 60 Fenwood Rd 1st Floor, Boston, MA 02115, United States; Department of Ophthalmology, Medical University of Warsaw, 61 Zwirki i Wigury Street, 02-091 Warsaw, Poland; Department of Ophthalmology, Medical University of Warsaw, 61 Zwirki i Wigury Street, 02-091 Warsaw, Poland; Nencki Institute of Experimental Biology, PAS, 3 Pasteur Street, 02-093 Warsaw, Poland; Department of Human Genetics, Radboud University Medical Center, Nijmegen, Geert Grooteplein-Zuid 10, 6525GA, The Netherlands; Department of Human Genetics, Radboud University Medical Center, Nijmegen, Geert Grooteplein-Zuid 10, 6525GA, The Netherlands; Department of Histology and Embryology, Medical University of Warsaw, 61 Zwirki i Wigury Street, 02-091 Warsaw, Poland; Department of Ophthalmology, Medical University of Warsaw, 61 Zwirki i Wigury Street, 02-091 Warsaw, Poland; Nencki Institute of Experimental Biology, PAS, 3 Pasteur Street, 02-093 Warsaw, Poland

**Keywords:** blind, cortical reorganization, low vision, lower visual field, upper visual field, visual cortex

## Abstract

In the visual cortices, receptive fields (RFs) are arranged in a gradient from small sizes in the center of the visual field to the largest sizes at the periphery. Using functional magnetic resonance imaging (fMRI) mapping of population RFs, we investigated RF adaptation in V1, V2, and V3 in patients after long-term photoreceptor degeneration affecting the central (Stargardt disease [STGD]) and peripheral (Retinitis Pigmentosa [RP]) regions of the retina. In controls, we temporarily limited the visual field to the central 10° to model peripheral loss. The central loss experienced by STGD patients led to an increase in RF size in the dorsal subdivisions of V1, V2, and V3. In contrast, peripheral loss in RP patients led to a bilateral increase in population RF sizes in V1 but a decrease in V2. Transient peripheral loss in controls led to an increase in RF size in V1 and a decrease in V2 and V3, regardless of the dorsal-ventral division of the cortical representation. Our findings suggest a dorsal-ventral difference in RF size in response to central visual field loss, likely reflecting the functional relevance of these divisions within the cortical representations of the visual field.

## Introduction

The early visual cortex is retinotopically organized, i.e. neighboring cortical regions respond to neighboring points in visual space. From early postnatal stages, the relationship between each neuronal receptive field (RF) size and its position within the visual cortical map remains stable; specifically, the smallest RFs are positioned in the areas representing the central retina, whereas the largest RFs are in the cortical areas representing the peripheral retina. These orderly maps are not static but remain malleable throughout life. While the RF size/cortical location relationship remains stable, RF sizes undergo dynamic changes throughout life. Evidence for such plasticity in mature sensory systems comes from experiments involving sensory deprivation or specific sensory stimulation of these systems in animal models and through human development (Thompson et al. 2017; Gomez et al. 2019). Such manipulations force cortical neurons to adapt to a new sensory environment and cause changes in their molecular composition and, consequently, their signaling and functional properties. We previously reported a specific dorsal neuroplasticity response after the induction of central retinal lesions in an animal model of macular degeneration (MD; Burnat et al. 2017). Here, we investigated the functional consequences of long-term central and peripheral visual field loss using population receptive field (pRF) mapping, a technique that allows the estimation of aggregate neuronal RFs within functional magnetic resonance imaging (fMRI) voxels (Dumoulin and Wandell 2008). This estimation relies on the analysis of fMRI activity recording during stimulation with dynamic stimuli, in this case, rotating wedges and expanding/contracting rings, which move over the visual field. This paradigm allows estimation of the pRF location in the visual field and therefore the eccentricity, i.e. the radial distance in visual degrees from the center of gaze (Sereno et al. 1995), as well as the size of pRFs (Dumoulin and Wandell 2008).

We aimed to compare reorganizations triggered by juvenile central loss of photoreceptors in patients with Stargardt (STGD) disease with matched onset of illness and duration to a group of retinitis pigmentosa (RP) patients with peripheral photoreceptor loss. The pRF modeling has been used previously to estimate the extent of cortical reorganization in patients with visual field defects, such as MD and STDG (Baseler et al. 2011; Haak et al. 2016; Ritter et al. 2019; Prabhakaran 2021; Pawloff et al. 2023), RP (Ferreira et al. 2016; Ritter et al. 2019), rod monochromatism (Baseler et al. 2002), glaucoma (Duncan et al. 2007; Prabhakaran et al. 2021), aging (Brewer and Barton 2014; Silva et al. 2021), hemianopsia (Papanikolaou et al. 2014, 2019), and choroideremia (Silson et al. 2018). Studies have shown that long-term central (*n* = 4; Masuda et al. 2008) and peripheral visual field loss (*n* = 3; Masuda et al. 2010) in V1 is task dependent. In MD patients with central photoreceptor degeneration, there are only two reports of changing pRF size and, these studies have only shown such an effect within the calcarine sulcus in V1, where an increase in the mean pRF size with a shift toward peripheral locations has been reported (*n* = 16, Baseler et al. 2011; *n* = 8 Ritter et al. 2019).

Until now, cortical reorganization in these groups of patients has been studied with small sample sizes which did not allow to perform the statistical comparisons within dorsal-ventral divisions of cortical representation or between visual areas V1, V2, and V3.

We studied two groups of patients with vision loss caused by genetic conditions: one with peripheral vision loss due to RP (*n* = 23) and with central vision loss due to STDG disease (*n* = 21). RP is an inherited condition affecting about 1 in 5,000 people worldwide, with the initial symptoms appearing in young adulthood (Cross et al. 2022). STGD is the most common hereditary cause of juvenile MD, affecting about 1 in 10,000 individuals (Cremers et al. 2020). RP is linked to mutations in around 90 genes, while STGD is mainly caused by a recessive mutation in the *ABCA4* gene (Ścieżyńska et al. 2016; Cremers et al. 2020).

To differentiate between transient and long-term peripheral visual field loss, we induced 15 min of transient binocular peripheral loss in a control group via non-translucent goggles with central holes to simulate the peripheral photoreceptor loss that occurs in RP. To our knowledge, until now, there is only one study that has tested the effects of peripheral visual field restriction in control subjects, but this peripheral limitation was performed by masking peripheral parts of the screen and simultaneously with fMRI signal (Prabhakaran et al. 2020). Here, for the first time, we conducted pRF mapping in a substantial cohort of patients with adequate cortical responses, enabling statistical comparisons with control subjects possessing full visual function. Analyses were performed separately for the lower and upper visual field representations within the dorsal and ventral subdivisions of the V1, V2, and V3 areas.

## Materials and methods

### Procedure

The MRI procedure was conducted at the Laboratory of Brain Imaging (Neurobiology Center, Nencki Institute of Experimental Biology). For the control group, the procedure was first performed in the unrestricted vision condition. Then, we temporarily blocked the peripheral visual field using swimming goggles with lenses that had been replaced; specifically, white opaque lenses with an aperture of 1.4 mm that limited the visual field to the central 10° were used. We used a set of 14 pairs of goggles with spacings ranging from 58 to 72 mm between the holes to account for the individual’s interocular distance (Ninghetto et al. 2024). During the 15-min break before scanning, the subjects were allowed to walk around freely while wearing the googles, and then the procedure was repeated. To rule out effects of the order of the procedure (ie unrestricted vision condition first, followed by limited vision condition), we tested limited vision first followed by the unrestricted vision condition in a subset of 12 of the controls, no significant differences were found (*t* = 4.875, *P* = 1.084; before: 3.04 ± 8.89; after: 2.97 ± 8.80). The results presented in this article are part of a larger study in which diffusion tensor imaging (DTI) and another fMRI protocol (Ninghetto et al. 2024) were performed as a result prolonging the total time inside the MRI per person to a maximum of 90 min.

### Participants

All participants were naive to the aim of the study, underwent routine ophthalmological examinations and reported no history of psychiatric or neurological disorders. Patient clinical and genetic data are listed in Supplementary Tables 1 and 2. Written consent was obtained from all participants. All procedures were performed following the relevant guidelines and regulations and were approved by the Ethical Committee, WUM (KB/65/A/2019).

### Inclusion criteria for patients

Forty-five RP patients with peripheral photoreceptor degeneration with tunnel vision presented a central residual visual field limited to a 10° diameter (Humphrey field analyzer), and best-corrected visual acuity equal to or superior to 20/40 (Early Treatment Diabetic Retinopathy Study; see Supplementary Table 1 for uncorrected acuity). Clinical examination of patients with RP revealed that the optic disk pallor and pigmentary deposits extended throughout the retina and narrowed blood vessels. Full-field flash electroretinography (ERG; RETIscan, Roland Consult, Germany) showed that the rod responses were more severely diminished than the cone responses, as in rod–cone dystrophy and their multifocal ERG (mfERG) results were abnormal. All RP patients were required to have their central 10 visual degrees to be functional. We tested this by determining which patients were not able to see the stimuli while wearing goggles and excluded the following: 1, 5, 6, 9, 12, 14, 20, 30, 31, 37, 38, 39, 40, and 45. RP patients 2, 15, 17, 18, 21, 22, 24, and 43 were excluded because they were not able to see the stimuli on the monitor and/or did not meet the inclusion criteria for undergoing MRI (eg having metallic implants in their body). The final RP group included 23 patients, aged 28 to 62 years, whose genetic testing was conducted using the RP-LCA smMIP platform, a targeted sequencing method that covers all currently known genes associated with RP (Panneman et al. 2023).

Twenty-four STGD patients with juvenile central photoreceptor degeneration presented central scotomas of 10 up to 20 degrees without foveal sparing and a best-corrected visual acuity equal to or superior to 20/40 (see Supplementary Table 2 for uncorrected acuity). Clinical examinations revealed a “bull’s eye” appearance of the macula. In flash ERG (RETIscan, Roland Consult), the full-field rod responses were normal, and the full-field cone responses were either normal or slightly reduced. mfERG revealed decreased responses in the central rings, suggesting abnormal function of the macula. Optical coherence tomography (Cirrus HD-OT Spectral Domain Technology, Zeiss, Germany) revealed a decreased thickness of the retina, most notably in the foveola. Patients 2, 13, and 15 were excluded because they were not able to detect the visual stimuli, and/or did not meet the inclusion criteria for MRI. The final group included 21 patients, aged 19 to 64 years, with STGD resulting from two *ABCA4* mutations (Cremers et al. 2020).

*Inclusion criteria for controls*

Forty-five healthy controls with normal or corrected-to-normal visual acuity aged 20 to 63 years (25 females; age 36.62 ± 12.52) were tested. Controls were matched to the RP and STGD patients for age and sex.

### fMRI acquisition

fMRI data acquisition was performed using a 3-Tesla MRI scanner (Siemens Magnetom Trio TIM, Erlangen, Germany) with a 12-channel phased-array head coil. For each participant, we acquired one structural T1-weighted image (repetition time = 2,530 ms, echo time = 3.32 ms) and evoked blood oxygenation level-dependent (BOLD) responses using a T2*-weighted gradient echo-planar imaging sequence (repetition time = 2,500 ms, echo time = 28 ms, flip angle = 80°, field of view = 504 × 504, and 72 axial slices with 3 mm slice thickness with no gap between slices). Stimuli generated in MATLAB (http://psychtoolbox.org/) were presented on a 32” LCD rear-projection screen with 1,920 × 1,080-pixel resolution, an active area of 69.8 × 39.3 cm (39.9 × 23.06 visual degrees at a viewing distance of 75 cm), and a refresh rate of 120 Hz (BOLD Screen 32, Cambridge Research Systems). The stimuli had a maximum radius of 9.2°, covering a total diameter of 18.4°, stimulus position remained stationary for the duration of one volume acquisition (i.e. 2.5 s) before shifting to the next position, enabling estimation of the spatial sensitivity of voxels. We used two types of stimuli: rotating wedges and expanding rings built from high-contrast checkerboards; stimuli rotated or expanded in a phase-encoded manner and each had two runs and four cycles per run. For each run, we acquired 97 volumes. First, we presented expanding rings, which increased in radius from fixation to the outer edge of the stimulus extent. The ring’s aperture was one-fourth of the maximum stimulus radius. Second, rotating wedges in counterclockwise rotation; the wedge angle was 45° and rotated around the fixation point to stimulate the full range of polar angles. This procedure followed the paradigm for the estimation of RF location and size across the visual cortex areas (Dumoulin and Wandell 2008).

**Fig. 1:**
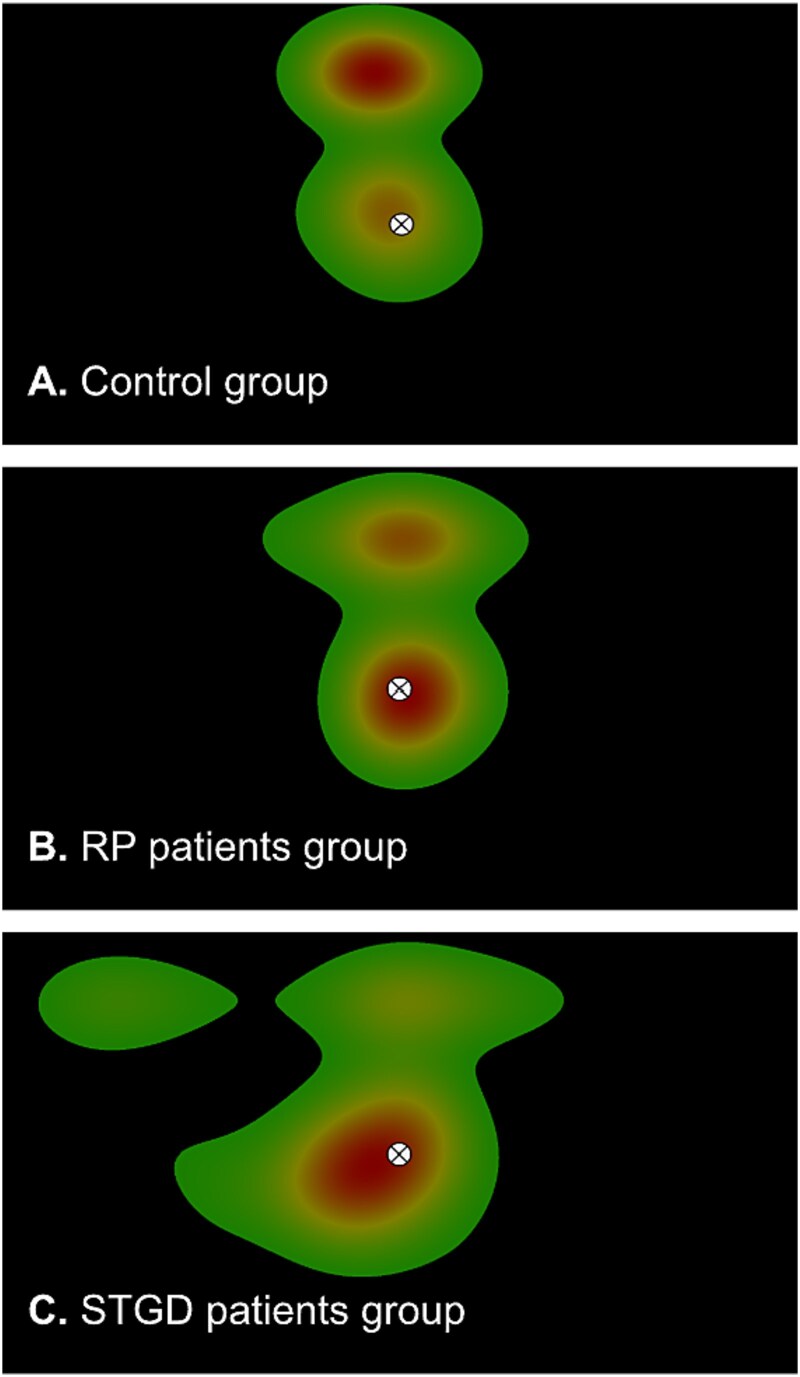
The mean fixation heatmaps during pRF mapping for: A) control participants, B) RP patients, and C) STGD patients. Mean fixation heatmaps are superimposed on the background, with a central circle depicting the fixation point. A “blob” with darker area represents the point where mean fixations lasted longer. Despite all the effort to set and calibrate the camera at its best, for A) controls and B) RP patients some of the subjects’ recordings shifted the real center of the screen to a higher location, this resulted in a heatmap with two centers of mass: 1 in the real center of the screen (marked by fixation point), 2 a higher center of mass due to shifting in calibration in some trials. Eye movements were recorded with the EyeLink 1000 Plus eye-tracker (https://www.sr-research.com/eyelink-1000-plus).

During the scanning session, we asked the participants to press a button on a response pad every time the fixation point would change its color at random intervals, alternating between red and green; unfortunately, we were not able to use this measure for all the patients, since some of them, together with other symptoms due to their eye disease, were not able to detect changes in colors. Since eye-tracking data were recorded concurrently with the fMRI protocol, we were able to aggregate fixation data for each group to generate fixation heatmaps, visualizing average eye positions throughout the scanning session (Fig. 1). These heatmaps confirmed that fixation patterns were similar between control participants and RP patients (Fig. 1A and B). Notably, the heatmaps for patients with STGD disease (Fig. 1C) revealed a single prominent fixation cluster centered around the fixation point.

### Image preprocessing

We used the FreeSurfer image analysis suite (http://surfer.nmr.mgh.harvard.edu/) to perform cortical reconstruction and volumetric segmentation of the T1-weighted anatomical scan and preprocessed the functional images with SPM12 (https://www.fil.ion.ucl.ac.uk/spm/) to correct for distortions and motion artifacts, coregister the anatomical image to the mean functional image, segment the coregistered structural image with the default tissue probability maps, and normalize the data to the Montreal Neurological Institute (MNI) space. Movement regressors were incorporated into the design matrix using the ARtifact detection Tools (ART) toolbox to identify and exclude motion-affected volumes exceeding a threshold of 3 mm and a rotation threshold of 0.05 radians.

We initially planned to adopt the settings used in the pioneering study by Dumoulin and Wandell (2008). However, due to the extended total duration of the scanning session, we had to reduce the time spent in the scanner, particularly for the patient cohort. Our full project included a recently published fMRI study by Ninghetto et al. (2024) and a DTI session which has not yet been fully analyzed. While Dumoulin and Wandell used six cycles, but with only six participants, our study included approximately four times as many patients and seven times as many healthy controls, although with only four runs. This adjustment allowed us to conduct the necessary statistical analyses between conditions. We included all participants in our analysis, even those with a low number of voxels, treating them as a random factor in our general linear model (GLM) design. By doing so, we improved the generalizability of the results and ensured that our analysis accounted for variability in the population while controlling for individual differences.

**Fig. 2:**
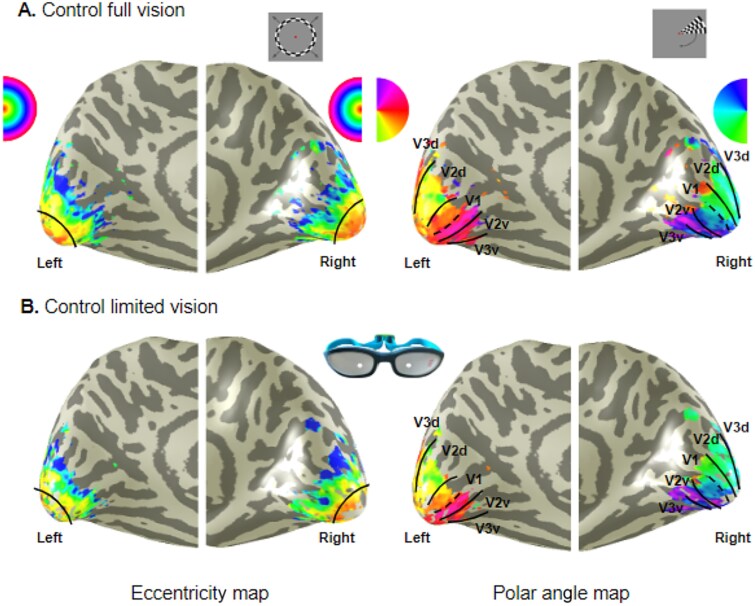
Polar and eccentricity maps shown on an inflated brain of a control participant #33 with full vision A) and while wearing goggles that limited their peripheral vision B) Retinotopic maps of eccentricity, shown in the left panels, were obtained from the expanding ring stimulus shown at the top. The color gradient from yellow to blue depicts central to peripheral eccentricity locations, as shown by the color wheel. The black line in A and B left panels, depicts a foveal representation of the visual field. The polar maps shown at the right panels are obtained with the wedge stimulus that rotated in a counterclockwise direction shown at the top. The borders of V1, V2, and V3 are mapped by reversals in polar angle color gradients. The separation of V1 dorsal and ventral regions by the calcarine sulcus is shown with a dashed line.

**Fig. 3:**
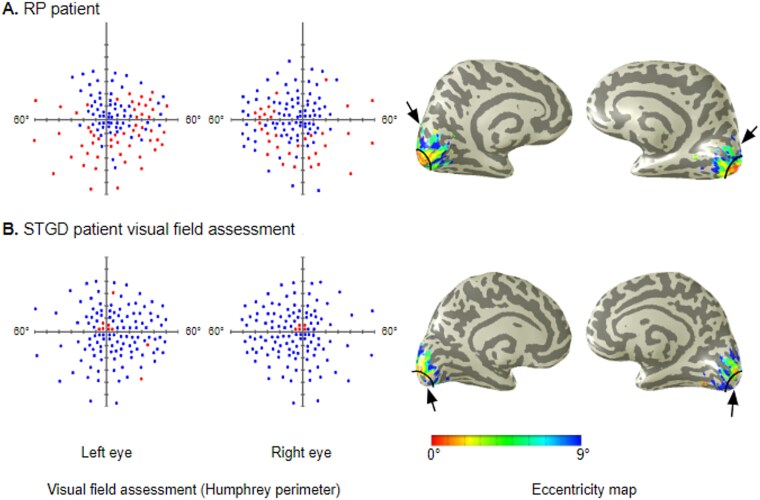
Representative inflated brains overlayed with eccentricity activation maps from RP patient #1031 A) and STDG patient #1395 B). The Humphrey perimeter test results for the left eye and right eye and the eccentricity maps for the inflated brain. Humphrey results are shown from the full-field 120-point screening, with blue (darker color) rectangles indicating stimuli that were seen and red (lighter color) rectangles indicating stimuli that were not seen by the participant. The arrows point to portions of the cortical maps representing the affected part of the visual field: A) peripheral locations for the RP patient; B) the representation of the fovea for the STGD patient. The ophthalmologic and genetic data for RP #1031 and STGD #1395 patients are listed in Supplementary Tables 1 and 2, respectively. Other visual elements are presented as in Fig. 2.

Finally, polar and eccentricity maps were obtained (Fig. 2A and B). We estimated pRF size and eccentricity in mrVista (Vista Lab, Stanford University) via a 2D-Gaussian pRF model and applied a threshold for including only voxels with an explained variance above 15%. For the limited vision condition, the pRF estimates outside the central 10° of the visual field were masked out from the results to remove any additional noise from outside the goggles’ aperture borders. As shown in Fig. 3, where we show the inflated brains of one RP patient and one STGD patient, the activation maps are highly different and exhibit several unresponsive spots, making manual delineation of regions of interest (ROIs) inappropriate.

**Table 1:** Main effects and interactions for the performed GLM analysis:

Mean pRF size	Main factor	*F* factor	*P*-value
**Area**			
Full vision vs limited vision	Group	*F*(1,402,314) = 36.0	<0.001
	Hemisphere	*F*(1,402,314) = 1,033.4	<0.001
	Area	*F*(9,402,314) = 1,202.3	<0.001
Full vision vs RP	Group	*F*(1,277,439) = 36.5	<0.001
	Hemisphere	*F*(1,277,439) = 989.7	<0.001
	Area	*F*(9,277,439) = 1,142.7	<0.001
Full vision vs STGD	Group	*F*(1,219,139) = 2,071.2	<0.001
	Hemisphere	*F*(1,277,439) = 989.7	<0.001
**dorsal/ventral and eccentricity**	Area	*F*(9,219,139) = 992.6	<0.001
Full vision vs limited vision	Main factor	*F*(1,402,254) = 43.1	<0.001
	Area	*F*(2,402,254) = 7,848.8	<0.001
	Hemisphere	*F*(1,402,254) = 631.2	<0.001
	Dorsal/ventral subdivisions	*F*(1,402,254) = 735.7	<0.001
	Bins nested in vision condition	*F*(66,402,254) = 1,122.2	<0.001
Control vs RP	Group	*F*(1,277,379) = 38.6	<0.001
	Area	*F*(2,277,379) = 5,760.3	<0.001
	Hemisphere	*F*(1,277,379) = 466.6	<0.001
	Dorsal/ventral subdivisions	*F*(1,277,379) = 178.1	<0.001
	Bins nested in vision condition	*F*(66,277,379) = 831.3	<0.001
Control vs STGD	Group	*F*(1,219,079) = 251.81	<0.001
	Area	*F*(2,219,079) = 857.51	<0.001
	Hemisphere	*F*(1,219,079) = 16.12	<0.001
	Dorsal/ventral subdivisions	*F*(1,219,079) = 792.08	<0.001
**pRF eccentricity shift**	Bins nested in vision condition	*F*(66,219,079) = 677.45	<0.001
Full vision vs Limited vision	Group	*F*(1,402,254) = 60	<0.001
	Area	*F*(2,402,254) = 12	0.000006
	Dorsal/ventral subdivision	*F*(1,402,254) = 62	<0.001
	Bins nested in vision condition	*F*(66,402,254) = 56,438	<0.001
Full vision vs RP	Group	*F*(1,277,379) = 21	0.00005
	Area	*F*(2,277,379) = 21	0.001
	Dorsal/ventral subdivision	*F*(1,277,379) = 75	0.001
	Hemisphere	*F*(66,277,379) = 39,669	0.001
Control vs STGD	Group	*F*(1,110,315) = 211	0.001
	Area	*F*(2,110,315) = 3	0.03516
	Dorsal/ventral subdivision	*F*(1,110,315) = 43	0.001
	Hemisphere	*F*(1,110,315) = 17	0.00003
	Bins nested in vision condition	*F*(66,110,315) = 31,181	0.001

**Fig. 4:**
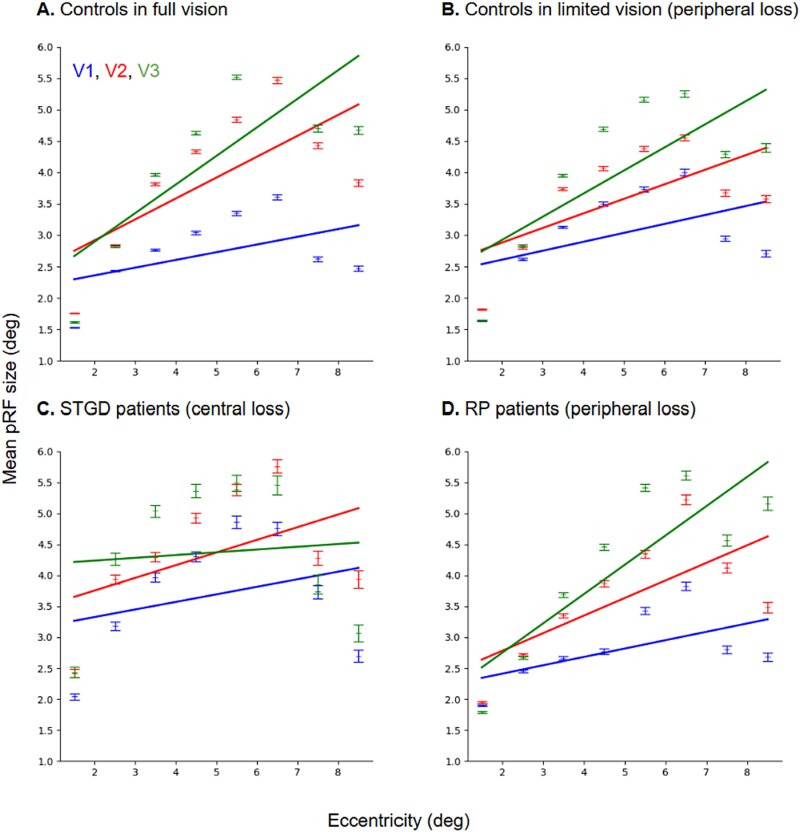
Mean pRF sizes in visual areas V1, V2, and V3 plotted as a function of eccentricity. A) Controls with full vision. B) Controls with limited vision. C) STGD patients. D) RP patients. Mean pRF size and standard error for visual areas: V1 (blue), V2 (red), V3 (green). The best fit is shown for each visual area. Note that in (A and B), the V3 data are overlaid for 1° to 2° of eccentricity.

Therefore, to delineate the ROIs for the V1, V2, and V3 areas and their dorsal/ventral subdivisions, we used the atlas from Wang et al. (2015). We divided the cortical representation of the visual field of each ROI into bins: 1° to 3°, 3° to 6°, and 6° to 9°; the 0° to 1° eccentricity was excluded from the analysis since it reflected the foveal representation, was prone to artifacts due to eye movements and was often difficult to activate sufficiently. Unfortunately, the signal-to-noise ratio in V3ab, V4, and V5+/MT areas was low, and retinotopic maps in these regions were incomplete, therefore our final analyses focused exclusively on V1 to V3. Eccentricities greater than 9° were also not analyzed to avoid potential pRF modeling inconsistencies close to the visible stimulus border. Although the final model returns an eccentricity for each area as a continuous variable (Fig. 4), bins were specified artificially, and their limits do not correspond to distinct, contiguous regions in the visual cortex.

### Statistical analysis

To investigate the differences in pRF size per area, we performed three separate nested GLM analyses for comparisons: (i) controls with full vision and controls with limited vision, (ii) RP patients with controls with full vision, and (iii) STGD patients with controls with full vision. We included the pRF size from individual voxels as outputs, and the participants were classified as a random factor, whereas groups, hemispheres, and the three visual areas were classified as fixed factors. The areas were nested into the group and hemisphere. We initially recruited two separate control groups: one matched for age and sex to the RP patients, and a second matched to the STGD patients. As there were no significant differences in age or sex between these two control groups were combined into a single control group.

To assess the correlation between the individual extent of the functional visual field, measured with Humphrey full-field perimetry, and the individual mean pRF size, we performed Pearson correlations between the percentage of “seen points” within the central 10° of the visual field and within a 60° radius of the visual field, between the left eye and the right pRF size in V1, V2, and V3, and between the right eye and the left pRF size for RP patients and for STGD patients separately.

**Fig. 5:**
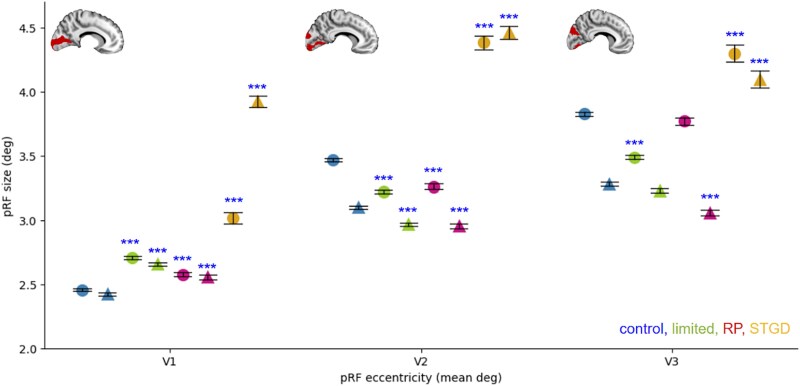
Mean pRF size increases for participants with peripheral and central visual field loss in V1 but not in V2 and V3 as measured within 1 till 9° eccentricity. A) pRF size in V1, B) in V2, and C) in V3. Note that in V2 and V3, pRFs decrease under peripheral loss and increase under central loss. Circles depict mean pRFs sizes in the left hemisphere and triangles the right hemisphere. At each ROI, blue color denotes controls in full vision (first and second markers; green, controls in limited vision (third and fourth markers); magenta, RP patients (fifth and sixth markers) and yellow, STDG patients (seventh and eighth markers). Whiskers denote the standard error. ^*^indicates significant difference within each hemisphere as compared with the control group in full vision; ^**^P < 0.0005, ^***^P < 0.00005.

**Fig. 6:**
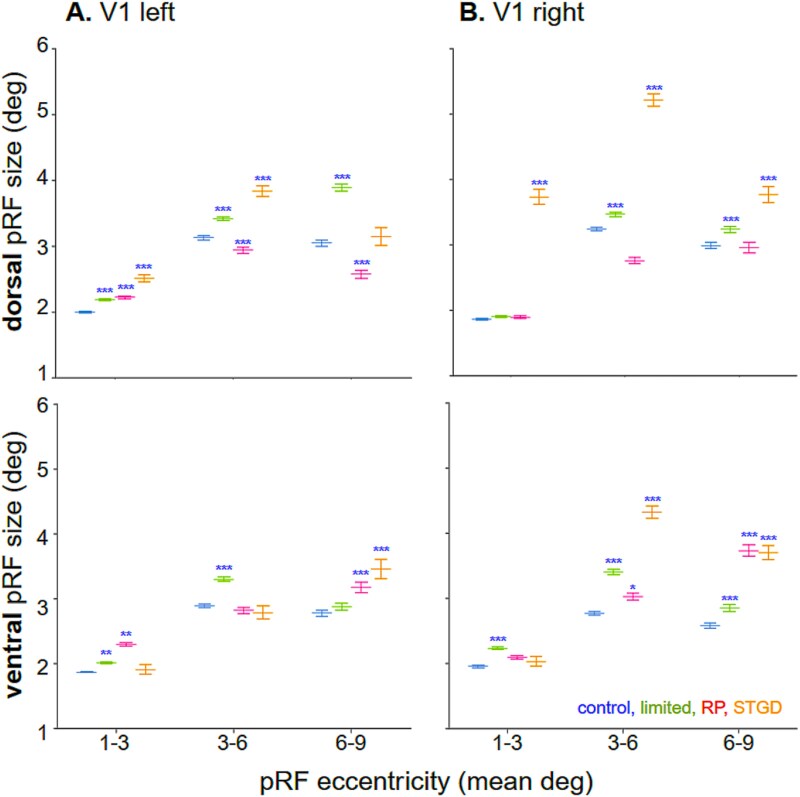
The mean pRF size in V1 increased after loss of the visual field. Mean data separated for A) left and B) right hemispheres in subjects with peripheral loss, ie limited peripheral vision in controls (green) and RP patients (magenta), and STDG patients with central loss (yellow). Significant changes after peripheral and central loss, relative to control subjects with full vision (blue). (Top panel) Dorsal visual hemifield. (Bottom panel) Ventral visual hemifield. Each result is shown separately for the eccentricity bins: 1° to 3°, 3° to 6°, and 6° to 9°. The significance compared with controls in full vision is indicated by ^**^P < 0.0005, ^***^P < 0.00005. Data are shown as means with standard error. Denotations as in Fig. 5.

**Fig. 7:**
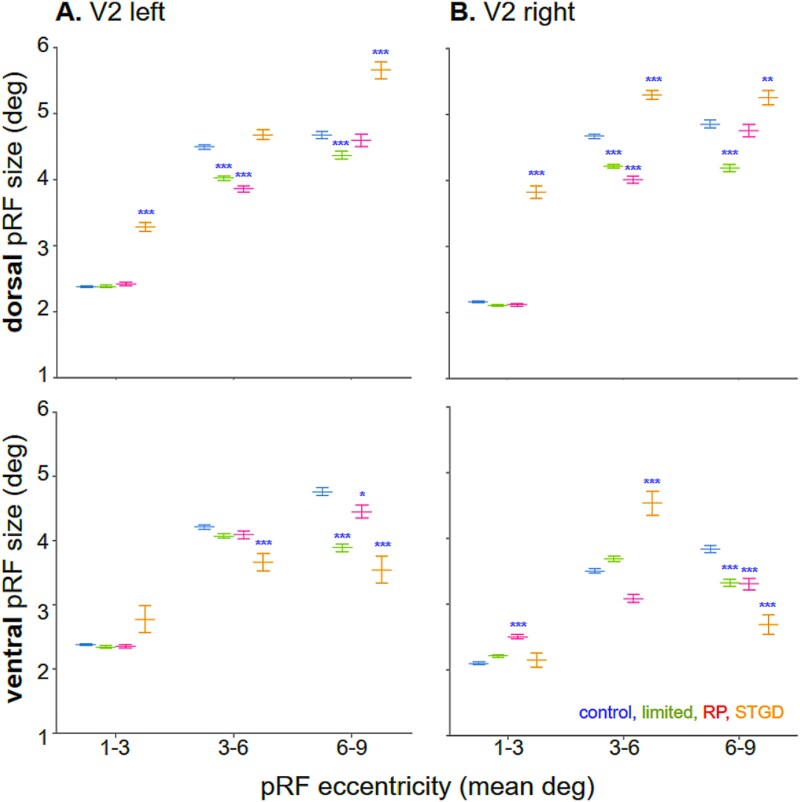
The V2 pRF size decreases in subjects after peripheral loss but increases after central loss. Mean data separated for A) left and B) right hemispheres in subjects with peripheral loss, ie controls with limited peripheral vision (green) and RP patients (magenta), and STDG patients with central loss (yellow). Significant changes after peripheral and central loss, relative to control subjects with full vision (blue). Note the increased pRF size in V2d for the STGD patients. Denotations as in Fig. 6.

**Fig. 8:**
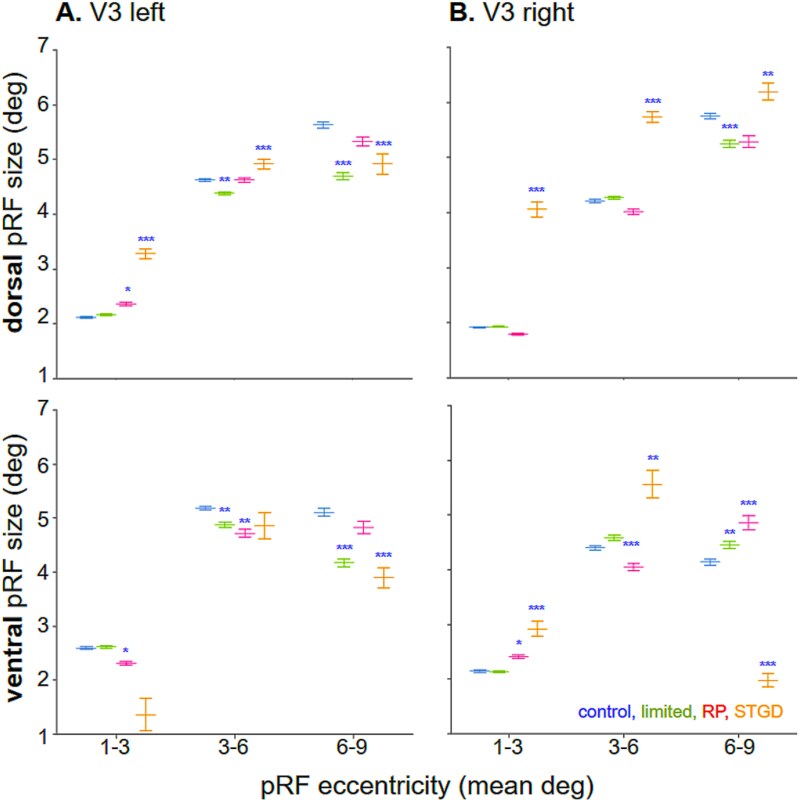
The V3 pRF size decreases in controls with limited peripheral vision and RP patients with long-term peripheral loss but increases in STDG patients with central loss. Mean data separated for A) left and B) right hemispheres in subjects with peripheral loss, ie controls with limited peripheral vision (green) and RP patients (magenta), and STDG patients with central loss (yellow). Significant changes after peripheral and central loss, relative to control subjects with full vision (blue). Notably, for the STGD patients, there was a bilateral increase in pRF size within the dorsal visual hemifield, but not within ventral V3 for the 1° to 3° and 3° to 6° bins. Denotations as in Fig. 6.

To investigate the differences between the dorsal and ventral subdivisions of areas (dorsal: V1d, V2d, V3d; ventral: V1v, V2v, V3v) and eccentricity bins, we performed three nested GLM analyses for comparisons: (1) controls with full vision and controls with limited vision, (ii) RP patients and controls with full vision, and (iii) STGD patients and controls with full vision. We included the pRF size from individual voxels; the participant identity was classified as a random factor, whereas group, hemisphere, bin, visual area, and dorsal/ventral subdivision were classified as fixed factors. The bins were nested into groups: visual areas, dorsal/ventral subdivisions, and hemispheres. Using the same GLM design, we investigated the shift in pRF location within the visual field using eccentricity as a dependent variable (Figs. 5–8). Descriptive statistics for pRF size and eccentricity are reported in Supplementary Tables 3 and 4. In the Results section, we report the post hoc results with Bonferroni correction for only significant main effects of all analysis. The all tested main effects of all interactions are listed in Table 1. The significance level was set at *P* < 0.05 and Statistica (1995–2020 TIBCO Software, Inc.) for all analyses.

## Results

First, we checked if the individual mean pRF size in the V1, V2, and V3 areas correlates with the individual extent of the patients’ loss of the visual field in RP or STDG patients using Pearson correlation separately between the percentage of “seen points” within the central 10° of the visual field or within a 60° radius of the visual field, and between the right eye and the left pRF size in V1, V2, and V3, and between the left eye and the right pRF size for RP patients and for STGD patients separately. This correlation was not significant (supplementary Table 5), therefore we present the further results based on the mean pRF, per each tested group of participants.

Next, to verify the pRF model applied on our data set, we calculated the mean pRF for each area, and divided the visual field for the 1° eccentricity bins to see if we could replicate well-established earlier (Dumoulin and Wandell 2008; Harvey and Dumoulin 2011) linear increase in the mean pRF size in V1, V2, V3 areas as a function of eccentricity (Fig. 4). Indeed, we could show for the controls with full vision (Fig. 4A), controls with limited peripheral vision (Fig. 4B), STDG patients (Fig. 4C), and RP patients (Fig. 4D), the increase of pRF size with increasing eccentricity. Similarly to results shown by Dumoulin and Wandell (2008), we noticed irregularities of the model in all tested groups of participants starting from 8 degrees, which were present in examined areas.

### In V1, the mean pRF size increases in all groups compared to controls with full vision but in V2 and V3, it remains increased only in patients with central loss of visual field.

In V1, all groups presented a significant bilateral increase in pRF size compared with that of the controls with full vision (controls with limited vision, *P* < 0.001; RP patients: left *P* = 0.00004, right *P* = 0.00002; and STDG patients, *P* < 0.001; Fig. 5A). In contrast, in V2 patients, controls with limited vision and RP patients showed a bilateral decrease in pRF size (for all comparisons, *P* < 0.001), whereas in STDG patients, the pRF size remained bilaterally increased (*P* < 0.001, Fig. 5B). A similar pattern was observed in V3: the unilateral pRF size decreased for controls with limited peripheral vision (left, *P* < 0.001) and in RP patients (right, *P* < 0.001), and the bilateral pRF size increased in STDG patients (*P* < 0.001, Fig. 5C).

To further explore the difference in response measured as pRF size to the peripheral and central loss of visual field, we performed another GLM analysis that separated the dorsal and ventral subdivisions of the cortical representation of the eccentricities of the visual field. Now, we separated the central visual field into three eccentricity bins 1° to 3°, 3° to 6°, and 6° to 9° and we report data for each hemisphere separately.

V1 response to transient and long-term peripheral loss is present in dorsal and ventral divisions. Figure 6 shows the V1 pRF size distribution and mean (white circles) as compared to controls in full vision (blue bars) with controls in limited vision (green bars), RP patients (magenta bars) and STDG patients (orange bars).

In the 1° to 3° eccentricity, controls with limited peripheral vision showed a bilateral significant increase in the V1v (left *P* < 0.001; right *P* = 0.0030) and in the left V1d (*P* = 0.000002), whereas RP patients only showed decrease in the left hemisphere V1d (*P* = 0.00058) and V1v (*P* < 0.001).

In the 3° to 6° eccentricity, controls with limited peripheral vision showed bilateral increases in the dorsal/ventral subdivisions (V1d: *P* < 0.001, *P* = 0.0004 and V1v *P* < 0.001, *P* < 0.001, left/right, respectively). The RP patients showed an increase in only the right V1v (*P* = 0.020), but a decrease in the right V1d (*P* < 0.001).

In the 6° to 9° eccentricity, controls with limited peripheral vision showed bilateral increase of pRF size in V1d (left *P* < 0.001; right *P* = 0.0007) and increase in the right V1v (*P* = 0.00004). Whereas RP patients exhibited an increase in V1v bilaterally (*P* = 0.00001, *P* < 0.001), but a in the left V1d (*P* < 0.001), showing somehow similar dorsal/ventral response as at 3 to 6 eccentricity.

V1 response to the central loss in STDG patients is present only in the dorsal division. The pRF size within the dorsal subdivision of V1 significantly increased bilaterally in bins in the 1° to 3° and 3° to 6° bins (*P* < 0.001 for all comparisons); in the 6° to 9° bin, a decrease was present in the right (*P* < 0.001). In the 3° to 6° bin, pRF size increased on the right (*P* < 0.001) and in the 6° to 9° bin, it increased bilaterally (*P* = 0.00005, *P* < 0.001).

The pRF size in the V2 and V3 areas depends on the visual field loss location. Figure 7 shows V2 pRF size distribution and mean (white circles) as compared to controls in full vision (blue bars) with controls in limited vision (green bars), RP patients (magenta bars), and STDG patients (orange bars).

In V2, in response to peripheral loss, the pRF size decreased. In the 1° to 3° bin, the data of RP patients resembled that of controls with limited peripheral vision and did not show any significant changes in pRF size, except for increased pRF size in the right V2v (*P* < 0.001; Fig. 7). In the 3° to 6°, in V2d, the controls with limited peripheral vision showed bilaterally. In RP patients, the pRF size showed bilateral V2d pRF size decrease (*P* < 0.001) and in the right V2v (*P* < 0.001). In the 6° to 9°, the groups with peripheral loss exhibited a bilateral decrease in V2v (limited: *P* = 0.00001, *P* < 0.001; RP: *P* = 0.0219, *P* < 0.001). In the limited peripheral vision group, the pRF size also bilaterally decreased in V2d (*P* < 0.001).

In contrast, after central loss in STDG patients, the pRF size in V2 increases and only within the dorsal subdivision. As in V1, in the 1° to 3°, the STDG patients exhibited the same pattern of bilateral increases in pRF size, which was specific to the dorsal subdivision of the V2 area (*P* < 0.001). In the 3° to 6°, an increase in pRF size was shown in the right V2d and V2v (*P* < 0.001). In the 6° to 9°, the pRF size also increased bilaterally at V2d (*P* < 0.001, *P* = 0.00012). In V2v, a bilateral decrease was found (*P* < 0.001).

Figure 8 shows V3 pRF size means (white circles) compared to controls in full vision (blue bars) with controls in limited vision (green bars), RP patients (magenta bars), and STDG patients (orange bars).

In V3, in response to peripheral loss the pRF size decreased as in V2. In the 1° to 3°, the pRF size in RP patients decreased significantly bilateral in V3v (*P* = 0.0218, *P* = 0.0149) and in the left V3d (*P* = 0.0184). In the 3° to 6°, the controls with limited peripheral vision exhibited a decrease in pRF size in the left V3d and V3v (*P* = 0.0006, *P* = 0.0002, respectively). In RP patients, the bilateral decrease was present in V3v (*P* = 0.00002, *P* = 0.00064). In the 6° to 9°, the limited group showed bilateral decreases in V3d and V3v (*P* < 0.001), whereas RP patients showed decreases in the right V3d and V3v (*P* = 0.00026, *P* < 0.001).

Again, after central loss in STDG patients, the pRF size in V3 increases. In the 1° to 3°, the STDG patients exhibited the same pattern, with a bilateral increase in pRF size in V3d (*P* < 0.001) and an increase in the right V3v (*P* = 0.000011, Fig. 8). In the 3° to 6°, a pRF size increase was present in the right V3d and V3v (*P* < 0.001, *P* = 0.00063). In the 6° to 9°, the pRF size decreased in the left V3d (*P* = 0.00012) and in bilateral V3v (*P* < 0.001).

In STDG patients, the mean eccentricity pRF location shifts toward peripheral locations. In the 1° to 3° only in dorsal subdivision of V1, V2, and V3 areas pRFs shifted bilaterally toward peripheral locations: V1d (*P* = 0.0001, *P* < 0.001), V2d (*P* = 0.04, *P* = 0.000003), and V3d (*P* = 0.0007, *P* < 0.001). In the 3° to 6°, the pRFs shifted toward peripheral locations in the right V1v (*P* = 0.0008), bilaterally in V2d and V2v (*P* < 0.001; *P* < 0.001 and *P* < 0.001, *P* = 0.031, respectively). In the 6° to 9°, the pRFs shifted toward peripheral locations in V1d left (*P* < 0.001), V1v right (*P* < 0.001), V2v left (*P* < 0.001), and V3v bilaterally (*P* < 0.001). Only in V2d, the pRFs shift toward a more central location bilaterally (*P* < 0.001; *P* = 0.018).

## Discussion

We showed for the first time that the pRF size modeling reflects the magnitude of visual field stimulation, not only within the primary visual cortex, but also in V2 and V3. In patients with juvenile central vision loss (STGD), we observed an increase in pRF size in V1, V2, and V3, which is consistent with the previously described functional connectivity between these areas in MD patients (Haak et al. 2016). Previously, an increase in pRF size in V1 was shown in eight MD and eight STDG patients (Baseler et al. 2011). Furthermore, studies in animal models of central vision loss, such as that caused by retinal lesions and induced scotomas, have also demonstrated an increase in RF size in V1 (Pettet and Gilbert 1992; Giannikopoulos and Eysel 2006).

Here, we demonstrate that the increase in pRF size is also maintained in the V2 and V3 areas. Importantly, the size of our cohort enabled statistical comparisons between the dorsal and ventral subdivisions of the visual areas, revealing that the pRF size increase is confined to the dorsal subdivision, within the V1 and V2 areas and to some extent in V3. The dorsal visual cortex response to central vision loss in STDG patients provides functional validation of the dorsal cortical thinning in the STGD reported by our group (Sanda et al. 2018). Consistently, we previously reported specific activation of the dorsal cortex in an MD model (Burnat et al. 2017). Here, we showed enlargement of the dorsal pRF size within the central 1° to 9° of eccentricity for STGD patients; this activation in the cortical representation of the central visual field likely demonstrates plastic reorganization, where central pRFs respond to peripheral stimulation (Gilbert and Li 2012). Similarly, Baker et al. (2005, 2008) reported foveal V1 activation in response to peripheral stimuli in early-onset MD patients but only in patients with complete loss of vision in the central retina, like our STDG patient group. Visual stimulation of the lower peripheral hemifield has been described as the most powerful method for guiding attention or visual control of movements (He et al. 1996; Danckert and Goodale 2001). Partial covering of the lower visual field impairs visually guided behaviors (Critelli et al. 2021; Weber et al. 2021). In STGD patients, the acquisition of peripheral features in dorsal RFs may reflect their role in exploring the lower visual field in everyday life, as shown here with eccentric shifts in pRF locations in V1, V2, and V3.

In V1, V2, and V3, the processing of the central upper visual field occurs in ventral cortical areas, whereas the peripheral lower visual field is processed in dorsal areas (Wandell et al. 2007). This organization of lower and upper visual field cortical representations originates from the retina, where there is a higher density of retinal ganglion cells (~60%) in the lower quadrant of the visual field (Curcio and Allen 1990). The projections from the superior hemiretina serve as inputs to the cortical representation of the lower visual field, resulting in the overrepresentation of the lower visual field (Schein and de Monasterio 1987), which explains the functional relevance of the dorsal cortical representation shown here.

In patients with RP, long-term peripheral vision loss induces a similar pattern of pRF size increase, but this effect is restricted to V1, while pRF sizes in the V2 and V3 areas are significantly decreased. Importantly, in healthy controls, the response to transient peripheral visual field loss exhibits a comparable pattern across the dorsal and ventral subdivisions, with an increase in pRF size in V1 and a corresponding decrease in V2 and V3. In contrast, the only previous study on healthy subjects, where visual stimulation was restricted to 4° or 7° of eccentricity, reported no change in pRF size in the V1 area but observed an increase in pRF size in the V2 and V3 areas (Prabhakaran et al. 2020). We hypothesize that the difference in outcomes may be attributed to our experimental approach, where we completely removed peripheral visual input by having participants wear non-translucent goggles with centrally located 10° diameter holes for 15 min prior to and during scanning. This method effectively eliminated any peripheral stimulation, including reflections from the scanner bore. In contrast, Prabhakaran et al. (2020) introduced peripheral limitations during fMRI signal acquisition by placing a peripheral gray mask around a centrally presented checkerboard stimulus.

The evidence of the effects of longer and full visual input removal prior to the experimental session is supported by research in which monocular occlusion was performed for 10 min, and V1 reorganization and perceptual distortion were measured behaviorally (Dilks et al. 2009; Jamal and Dilks 2020). Most likely, these observed adaptations are not long-lasting, as shown by a study in which healthy adults experienced 4 d of blurred vision (Haak et al. 2015). The results of Haak et al. (2012) show that such an assumption is also valid for central loss. Consistent with our results in STDG patients, the removal of central stimulation in healthy participants resulted in an increase in pRF size and a shift toward peripheral locations in V1. Our findings show less significance for the measurements of the pRF eccentricity shift, which is generally in line with findings for V1 in eight STDG patients with peripheral eccentric pRF and more central pRFs in eight RP patients (Ritter et al. 2019).

We demonstrated that both RP patients with long-term peripheral loss showed a decrease in pRF size in V2 and V3 and controls experiencing temporary peripheral loss. The difference in the responses between V1 and V2/V3 may be attributed to a shift in attentional focus from the periphery to the center, induced by wearing the goggles, and peripheral loss of photoreceptors in RP patients. Recently, we demonstrated that under identical visual conditions and with the same participant cohort, both controls in limited vision and RP patients exhibit similarly reduced activation in MT+/V5, salience-processing areas, and the superior temporal cortex, compared to controls with full vision (Ninghetto et al. 2024). MT +/V5 is an early processing area within the dorsal visual pathway, playing a critical role in motion perception and attentional processes (eg Zeki et al. 1991). Womelsdorf and colleagues (2006) demonstrated that directed attention can influence RF size in alert monkeys, with shifts in attention leading to a reduction in RF size within MT. This suggests that attentional modulation can refine the spatial properties of RFs in extrastriate areas, as in V1 (Roberts et al. 2007). The differential size of pRF responses to the obstruction of peripheral visual input in V1 compared to V2 and V3, as described by us, may be explained by the disruption of top-down feedback from extrastriate areas to V1. Nurminen et al. (2018) demonstrated that feedback from V2 plays a critical role in regulating RF size and neuronal responses in V1. In conditions where this feedback is diminished, such as in visual field loss, V1 exhibits an increase in RF size, likely compensating for the loss of peripheral input. In contrast, the same disruption of feedback mechanisms can lead to reduced responses in higher visual areas like V2 and V3, which depend not only on feedforward input from V1 but also on top-down modulation from extrastriate cortices. Similarly, optogenetic inactivation of connections between V1 and V2 in the marmoset brain has been shown to result in increased RF sizes in V1 and reduced activation in V2 (Angelucci and Bullier 2003).

We must note a limitation of our findings: the results cannot be directly translated to clinical settings, as we did not find a significant correlation between individual pRF sizes and the ophthalmological measurements of low vision parameters available for our patient cohort. This lack of correlation suggests that additional factors, beyond the ophthalmological metrics we measured, may be influencing the observed changes in pRF size. As in most patient studies, variability in data quality was observed. Therefore, we report only reliable data from visual areas V1 to V3, as consistent measurements could not be obtained for areas V4 and V5.

In conclusion, our findings demonstrate that the response to visual field loss depends on dorsal-ventral cortical subdivisions and provide valuable insights into the neural mechanisms underlying both transient and long-term visual field loss, supporting previous work (Burnat 2015). Notably, the observed similarity in pRF changes between patients with RP and controls experiencing transient peripheral visual loss suggests that transient deprivation may predict long-term cortical adaptations. Furthermore, we have characterized visual processing alterations associated with visual field loss, aiming to stimulate future research into spatially targeted visual field stimulation as a potential rehabilitation intervention. Such studies could deepen our understanding of cortical reorganization mechanisms and guide the development of targeted strategies to optimize visual function in individuals with visual field impairments.

## Supplementary Material

Supplementary1_CerCor_Ninghetto_bhaf237

Supplementary2_CerCor_Ninghetto_bhaf237

Supplementary3_CerCor_Ninghetto_bhaf237

Supplementary4_CerCor_Ninghetto_bhaf237

Supplementary5_CerCor_Ninghetto_bhaf237

## Data Availability

Ophthalmological and pRF mapping results and scripts for pRF data analysis will be made available to clinicians or researchers upon reasonable request.
